# Supplementary material for: A Method for High‐Throughput Measurements of Viscosity in Sub‐micrometer‐Sized Membrane Systems
Source: Chembiochem. 2019 Dec 2;21(6):836–44. doi: 10.1002/cbic.201900510 (PMC7154536; doi:10.1002/cbic.201900510)
Supplement: Supplementary file 1 — Supplementary [file CBIC-21-836-s001.pdf]

Author Contributions

G.C. Conceptualization: Lead; Data curation: Equal; Formal analysis: Equal; Investigation: Equal; Methodology: Equal; Software: Equal; Validation: Equal; Visualization: Equal; Writing - Original Draft: Equal; Writing - Review & Editing: Equal
E.P. Conceptualization: Supporting; Data curation: Equal; Formal analysis: Equal; Investigation: Equal; Methodology: Equal; Software: Equal; Validation: Equal; Visualization: Equal; Writing - Original Draft: Equal; Writing - Review & Editing: Equal
J.S. Conceptualization: Supporting; Funding acquisition: Lead; Investigation: Supporting; Project administration: Lead; Resources: Lead; Supervision: Lead; Writing - Original Draft: Supporting; Writing - Review & Editing: Supporting.
